# Health Behaviors and Associated Feelings of Remote Workers During the COVID-19 Pandemic—Silesia (Poland)

**DOI:** 10.3389/fpubh.2022.774509

**Published:** 2022-01-27

**Authors:** Agnieszka Białek-Dratwa, Elżbieta Szczepańska, Mateusz Grajek, Beata Całyniuk, Wiktoria Staśkiewicz

**Affiliations:** ^1^Department of Human Nutrition, Department of Dietetics, Faculty of Health Sciences in Bytom, Medical University of Silesia in Katowice, Zabrze, Poland; ^2^Department of Public Health, Department of Public Health Policy, Faculty of Health Sciences in Bytom, Medical University of Silesia in Katowice, Zabrze, Poland; ^3^Department of Food Technology and Quality Evaluation, Faculty of Health Sciences in Bytom, Medical University of Silesia in Katowice, Zabrze, Poland

**Keywords:** eating behavior, well-being, emotional eating, remote work, COVID-19

## Abstract

During the COVID pandemic in Poland, lockdown and remote work affected a very large segment of the population. This situation has many negative consequences both in terms of health and also emotionally. In our study, we focused on eating behaviors as well as health behaviors such as alcohol consumption, smoking, physical activity while working remotely, but also the emotions that occur while being at home working for long periods of time and how these emotions affect diet, eating behaviors and overall well-being using the standardized WHO-5 and TFEQ13 questionnaires. Surveys completed by 225 individuals doing remote work from home or hybrid work. During lockdown and remote work, 64.4% people noticed changes in eating behaviors: of which 44.0% people eat more than before lockdown, while 20.0% eat less than before; 36.0% believe they did not notice a change regarding the amount of food consumed. Changes in eating behavior did not correlate with body weight (*p* = 0.37), but did correlate with changes in body weight (*p* = 0.00000). Body weight correlated with changes in body weight that occurred in the study group during lockdown (*p* = 0.000004). Individuals who restrict eating according to TFEQ 13 are more likely to report well-being (WHO-%), whereas individuals who are observed to lack control over eating and eat under emotional duress are more likely to report poor well-being (*p* = 0.000000). The study confirmed the change in dietary behavior and the occurrence of adverse health eating behaviors among remote and hybrid workers during the COVID-19 pandemic.

## Introduction

COVID-19 (coronavirus disease) caused by coronavirus has caused millions of infections but also deaths worldwide (1–3). The first cases in Poland took place in March 2020. By 19.07.2021, 2,881,491 people in Poland had become ill with SARS-CoV-2 and 75,215 had died (4). Since the beginning of the pandemic, all communities were forced to take measures to reduce the number of infections. Strategies were implemented to delay the spread of the virus in order to reduce the burden on health care workers and protect those most susceptible to infection (5). The actions taken by individual countries varied at different stages of the pandemic. In general, social isolation and personal protective measures were recommended everywhere. As a result, approximately 4 billion people worldwide were forced into home isolation. The recommendations often forced changes in work activities. Lockdown was associated with various forms of reduced contact in the work process. Several forms of occupational restriction were observed, e.g., reduced working hours, working from home, hybrid model, rotational work. The workplace of many people has moved to their own homes (home office). The hybrid model of work is that some employees remain at work in the office and some work remotely from home. During the lockdown there was a rotation of those working in the office worked from home and those working at home office worked in the office. Such a model of work was introduced in Poland, among others, by the Regulation of the Minister of Health on establishing certain restrictions, orders and bans in connection with the occurrence of an epidemic on the basis of the Act of 5 December 2008 on preventing and combating infections and infectious diseases in humans. Subsequent waves of COVID-19 infections in Poland caused that some sectors of the economy were completely blocked for a dozen or so weeks, e.g., the gastronomy sector, cosmetic and hairdressing services, and other sectors, especially office work, were completely relocated in the remote system called home office, e.g., education, higher education, clerks, office workers (6–8).

These activities resulted in new job responsibilities, the need to learn new skills such as technical skills. They also often necessitated the need to combine remote work with childcare and assisting children with remote schooling. As experienced in COVID-19, home isolation and especially mandatory quarantine can result in lowered mood, irritability, emotional disturbance, exhaustion, and feelings comparable to emotional disturbance and depression (8).

Before the outbreak of the pandemic, remote working in most European countries was experienced by about 10% of employees. In a highly technological country like Japan, remote working was only used by 16% of workers (9). At the time of the pandemic, at least four billion people were confined worldwide, working remotely, teaching children at home, and facing the challenges of quarantine and stressful events (8).

COVID-19 outbreaks have been documented among office workers in many countries, including banks, corporate headquarters, government offices, and call centers, and thus decisions have had to be made to forcibly send office workers to remote or hybrid work (10, 11).

The COVID19 pandemic—prompts reflection on the implications it may have on society. The spreading disease and actions taken by individual governments affect every sphere of life (12, 13). Probably one can distinguish quite a few benefits of working from home especially in the global sphere e.g., economic, convenience limiting commuting time to zero. Remote work is also about health risks and consequences that result from lifestyle changes. Health behaviors are one of the most important lifestyle components and determinants of health. Proper nutrition and general lifestyle hygiene are the foundation of keeping the body in the best possible mental and physical condition. Neglect in this regard is one of the causes of health disorders (8, 14).

Activities that limit the activity of individuals have led to decreased physical activity and dietary changes that can accelerate sarcopenia, deterioration of muscle mass and function, and increased body fat (14). These changes in body composition correlate positively with the development of many chronic diseases such as cardiovascular disease, diabetes, osteoporosis, also disease symptoms such as weakness, cognitive decline and depressive symptoms (14). Home isolation associated with the COVID-19 pandemic as a stressful situation may affect eating habits that can induce both hypophagia and hyperphagia, as well as compulsive overeating leading to weight gain by eating extra meals called snacks, consuming more alcohol (8, 15, 16). An individual well-balanced diet rich in phytonutrients with immunomodulating effects may play an important role in the response to infectious agents. Nutritional deficiencies are associated with a higher susceptibility to viral infections and a more severe clinical course of the disease (8).

The persistent pandemic condition necessitates determining the long-term impact of the pandemic on behavior and identifying effective strategies to support families in the context of COVID-19 (17). Lifestyle studies conducted in many countries, in the era of the pandemic, indicate the need for monitoring the problem and extensive population-based studies (13, 17, 18).

## Materials and Methods

### Test Sample

The survey was conducted in March and April 2021, among people working remotely, during another lockdown in Silesia (Poland) when all educational institutions for children and young people (nurseries, kindergartens, elementary school, secondary schools), universities were closed, some office work was done in a hybrid system or in a complete home office system.

The questionnaire was distributed to medium-sized companies (employing <250 in a fiscal year) in the Silesian region that, as a result of lockdown, were forced to switch to a remote work mode or use IT tools to some extent to reduce the possibility of SARS-CoV-2 virus transmission. The study used CAWI—Computer-Assisted Web Interview to reach the specific study group during the pandemic and lockdown to ensure safety during the survey. The return rate of the questionnaire at this stage was estimated at 13%.

In the study, the following demographic data were obtained: gender, age, education (primary, vocational, secondary, tertiary) and body weight and height and on this basis Body Mass Index (BMI) was calculated and interpreted based on WHO recommendations for adults: <18.5 kg/m^2^–underweight, 18.5–24.9 kg/m^2^–normal weight, 25.0–29.9 kg/m^2−^ overweight, 30.0–34.9 kg/m^2−^ grade I obesity, 35.0–39.9 kg/m^2^–grade II obesity, >40 kg/m^2^ grade III obesity (19).

The levels of education adopted in the survey were established in accordance with Polish legal regulations: primary education, secondary education (high school) and higher education (university).

### Study Inclusion and Exclusion Criterion

Inclusion criteria for the study were: age of majority (over 18 years old), informed and voluntary consent to participate in an anonymous study, correctly completed questionnaire, remote work performed from own home office in minimum of last 30 days in hybrid or total home office. The criteria for exclusion from the study were age below 18 years, performing work from the employer's premises, and non-working status due to lockdown of the workplace. The above criteria were verified by answering the survey questions regarding the form of work during the pandemic. Moreover, as previously mentioned, all respondents were employees of companies located in the Silesian region.

### Research Tool

The research tool was a survey questionnaire that consisted of several parts.

In the first part, the questionnaire used in the study assessed:

1. form of work during lockdown (remote work from home, hybrid work—alternating work from home along with work from place of employment, work at place of employment), time spent working remotely, comparison of remote work time to traditional work time before the onset of the SARS-COV2 pandemic;
2. current weight and height and the change in weight during remote work;
3. dietary behaviors on the basis of the following scales: frequency of consumption of meals during the day, consumption of the first and second breakfast, frequency of consumption of selected products and amount of drunk liquids, which were related to the current dietary recommendations in Poland according to the National Center for Nutrition Education in 2020. (20, 21).
4. lifestyle through questions about the amount of sleep, amount of alcohol consumption and type of alcohol, smoking, and amount of physical activity each day.

To investigate the effects of prolonged home isolation while working remotely from one's home during the COVID 19 pandemic on the eating behaviors of Polish adults, an anonymous online survey was conducted, examining eating behaviors as well as verifying attitudes such as alcohol consumption, smoking, physical activity, and sleep. The study also assessed the emotional aspect of eating behavior using Karlsson's shortened version of the Stunkard and Messika TFEQ-13 scale (22–24) and the WHO 5 well-being index (25, 26).

The Three-Factor Eating Questionnaire-13 (TFEQ-13) was used in the second part of the survey. The questions in the TFEQ-13 questionnaires form three subscales:

1. Cognitive Restraint of Eating−5 questions (O1–O5),
2. Uncontrolled Eating−5 questions (R1–R5).
3. Emotional Eating−3 questions (E1–E3).

The TFEQ-13 questionnaires use standardized responses on a 4-point scale from 0 points to 3 points (definitely yes −3 points; rather yes −2 points; rather no −1 point; definitely no-−0 points). Question 13 (R5) was recoded as follows: 1 and 2–0 points; 3 and 4–1 point; 5 and 6–2 points; 7 and 8–3 points. Total points are calculated separately for each subscale. Values for the total scale are not calculated. A higher total score for a subscale indicates the severity of the disturbance in that subscale. The following statements in the TFEQ-13 were used:

1. I deliberately put myself on small portions of food to affect my weight.
2. I eat when I feel nervous.
3. Being in the company of someone who eats often makes me feel so hungry that I have to eat something too.
4. When I am sad, I often overeat.
5. When I see something delicious, I often get so hungry that I have to eat immediately.
6. I often get so hungry that my stomach seems bottomless.
7. I am constantly hungry and so I find it hard to stop eating until I have eaten everything on my plate.
8. When I feel lonely I comfort myself with food.
9. I consciously control the amount of food at a meal so I don't gain weight.
10. I refrain from eating certain foods because I get fat from them.
11. I am always hungry enough to eat at any time.
12. Are you likely to consciously eat less than you feel like eating?
13. How much do you restrict food? (Scale from 0 to 8 points −0 means I do not restrict at all, 8 means I always restrict).

Psychometric evaluation of the TFEQ-13 was done within three categories: food restriction (first component-−5 indicators/questions: 1, 9, 10, 12, and 13), lack of control over eating (second component −5 indicators/questions: 2, 5, 6, 7, and 11), eating under the influence of emotions (third component −3 indicators/questions: 2, 4 i 8). The values were calculated separately for each subscale. The higher the score was, the higher the intensity of restrictive eating, lack of control over eating, or eating under the influence of emotions was (22–24). The internal consistency of alpha-Cronbach's alpha for the whole scale in the adaptive study of the questionnaire was 0.78. In the own study, this index was estimated at 0.81, indicating high reliability of the analysis conducted with the scale^1^

The third part of the survey used the WHO Good Self-Concept Indicators Questionnaire 5 in Polish. To determine respondents' level of recent well-being (since transitioning to remote work). The WHO-5 questionnaire contained five items related to positive phrases: I felt cheerful and in a good mood, I felt calm and relaxed, I felt active and energetic, I woke up feeling fresh and rested, My daily life was filled with things of interest (25, 26). The respondent was asked to rate the extent to which each of the 5 statements applied to him/her, given the past 14 days. Each of the 5 statements was scored from 5 pts to 0 pts where 5 pts—All the time, 4 pts—Almost all the time, 3 pts—More than half the time, 2 pts—Less than half the time, 1 pts—Occasionally, 0 pts- Never. The raw score was obtained by summing the scores of all responses and ranged from 0 to 25, with 0 indicating the worst possible quality of life and 25 indicating the best possible quality of life. Scoring a certain point score (converted to percentages) determines the respondent's level of well-being. The raw score was multiplied by 4 to get the percentage score. A percentage score between 0 and 54% was considered to indicate poor well-being and a score between 55 and 100% was considered to indicate good well-being.

### Statistical Analysis

Statistical analysis was performed using the software in Statistica v. 13.1 (StatSoft Inc., Tulsa, OK, USA). Non-parametric tests were used for statistical analysis. Differences between groups were tested by Pearson's Chi-square test, with Fisher's exact, Yates, and the level of statistical significance was taken at *p* < 0.05.

### Ethical Consents

All study participants were informed of the purpose of the study, its anonymity, and were asked to accept the data sharing policy. Information about informed and voluntary participation in the study was at the beginning of the questionnaire. The study was approved by the Bioethics Committee of the Medical University of Silesia in Katowice (PCN/0022/KB/211/20) in light of the Act on Medical and Dental Professions of December 5, 1996, which includes a definition of medical experimentation.

## Results and Discussion

### Demographic Characteristics

A total of 272 individuals participated in the study, which is a representative sample on the assumption that Silesian region is inhabited by 2,916,981 people over 18 years of age (working age); however, due to not meeting the inclusion criterion for the study, surveys completed by 225 individuals doing remote work from home or hybrid work, including 79.1% women and 20.8% men, were included in the final data analysis. In the survey, 188 individuals (83.5%) had done remote or hybrid work since March 2020, 8 individuals (3.5%) since July 2020, 22 individuals (9.7%) since November, and 7 individuals (3.1%) had done remote work before the pandemic. Overall, 79.1% of the study group was female. The mean age of all subjects was 34.9 years ± 8.7. The mean BMI of the study group was 24.6 kg/m^2^ ± 4.8. (4.0%) women and 0 men were underweight. Normal body weight was 166 (65.1.1%) women and 17 (36.1%) men. 34 women (19.1%) and 19 (40.4%) men were overweight, 18 (10.6%) women and 11 (23.4%) men were obese. In the study group, 26(11.6%) people had secondary education and 199(88.4%) had higher education, 137(60.9%) did remote work from home and 88(39.1%) did hybrid work. 94 (41.7%) people spent 7–8 h per day on work and 101(44.8%) confirmed that remote work takes more time than traditional work. Demographic characteristics and those related to the nature of work during pandemic and lockdown are shown in Table 1.

**Table 1:** Study group by sex, age, weight, education, and form of work (*n* = 225).

**Gender**	**225 persons (100%)**
Female *n* (%)	178 (79.1%)
Male *n* (%)	47 (20.8%)
**The average age** ± SD (min-max)	34.9 years ± 8.7 (21–59 years)
**Body mass index BMI (kg/m**^**2**^**)** ± SD (min-max)	24.6 kg/m^2^ ± 4.8 (16.9–56.3 kg/m^2^)
Underweight (<18.5), *n* (%)	9 (4.0%) W: 9 (5.0%) M: 0(0.0%)
Normal weight (18.5–24.9), *n* (%)	133 (59.1%) W: 166 (65.1%) M: 17 (36.1%)
Overweight (25.0–29.9), *n* (%)	53 (23.6%) W: 34 (19.1%) M: 19 (40.4%)
Obese (≥30.0) *n* (%)	30 (13.3%) W: 18 (10.6%), M: 11 (23.4%)
**Education:** ***n*** **(%)**	
Primary	0 (0%)
Secondary (high school)	26 (11.6%)
Higher	199 (88.4%)
**Form of work:** ***n*** **(%)**	
Hybrid work (remotely from home alternating with	88 (39.1%)
Working at my place of employment)	
Work remotely from home	137 (60.9%)
**Hours per day devoted to remote working:** ***n*** **(%)**	
1–2 h	2 (0.8%)
3–4 h	33 (14.6%)
5–6 h	42 (18.6%)
7–8 h	94 (41.7%)
9–10 h	44 (19.5%)
11 and more h	10 (0.4%)
**Evaluation of remote working time**	
I only work remotely (no comparison possible)	10 (0.4%)
Less time than traditional work	55 (24.4%)
As much time as traditional work	59 (26.2%)
More time than traditional work	101 (44.8%)

### Changes in Body Weight and Eating Behavior During the COVID-19 Pandemic

In the study group, 125 (55.5%) subjects declared that their body weight increased while working remotely from March 2020: 95 (42.2%) people increased their weight by 1–5 kg, 28 (12.4%) people increased their weight by 6–10 kg, and 2 (0.9%) people increased their weight by 11–15 kg. Forty eight people (21.3%) stated that they had reduced weight since working remotely including 32 people (14.2%) 1–5 kg, 11 people (7.8%) 6–10 kg, 4 people (1.7%) 11–15 kg and over 16–20 kg-−1 person (0.4%).

**Figure 1:**
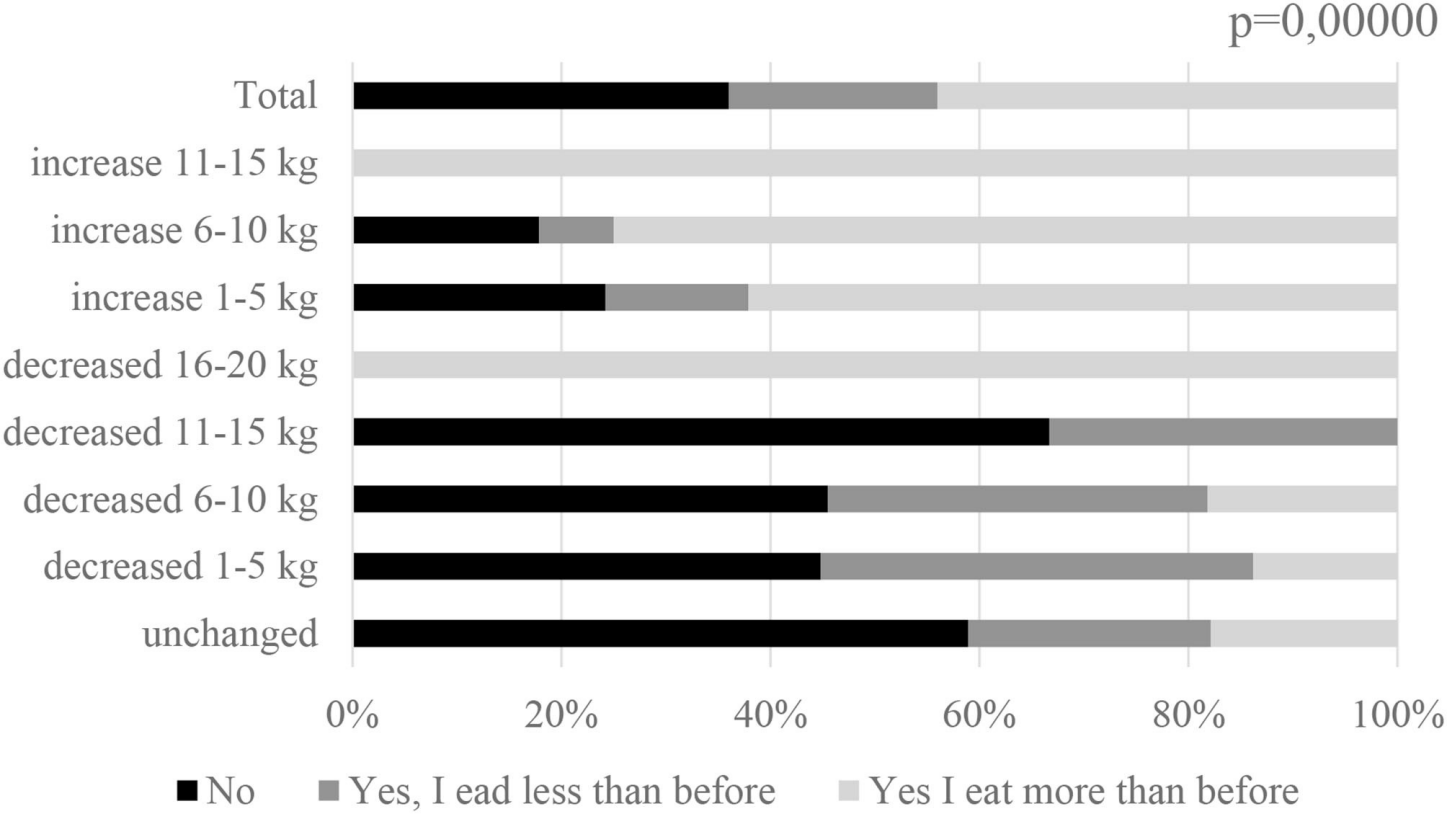
Change in eating behavior vs. change in body weight among subjects *n* = 225.

**Figure 2:**
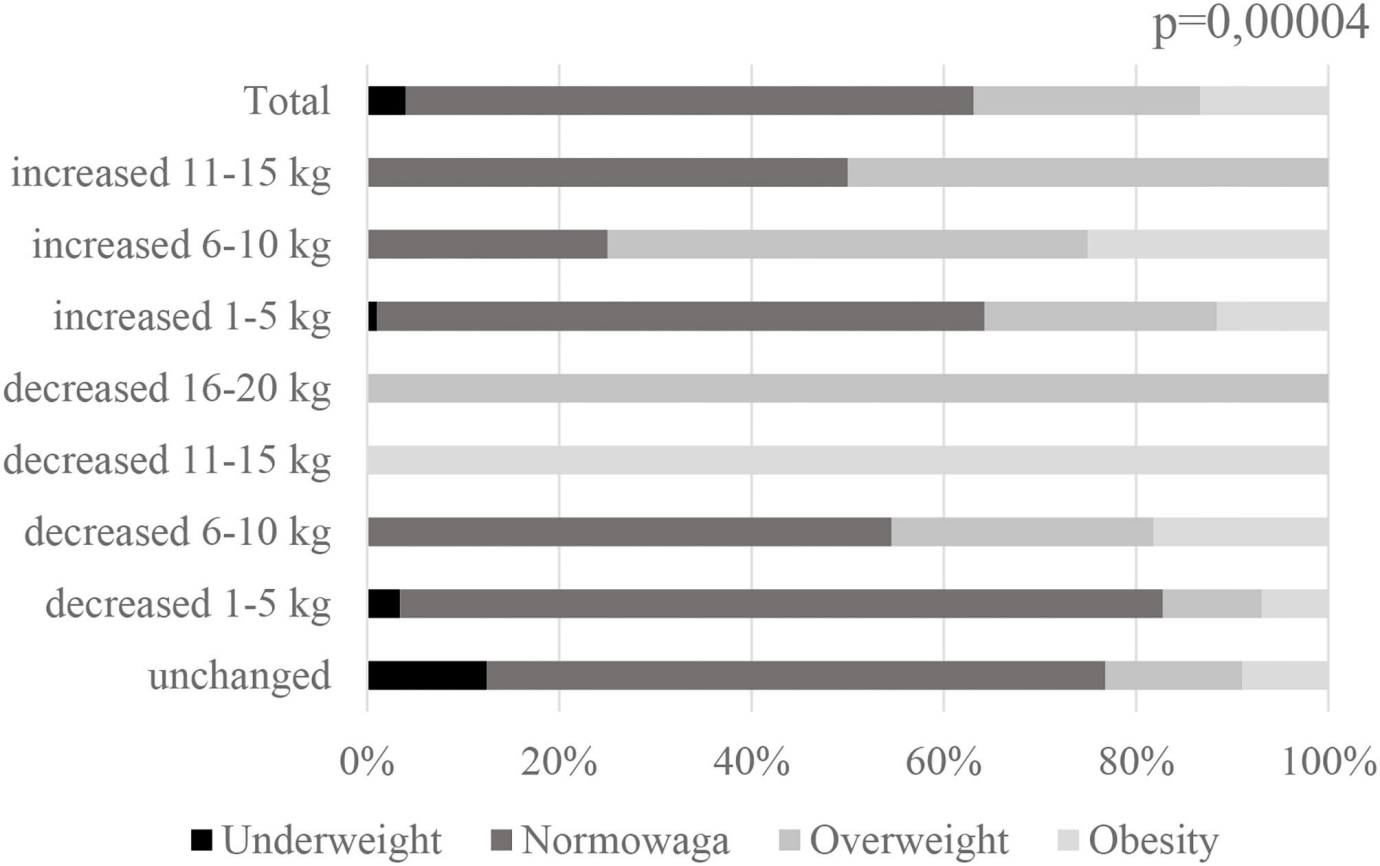
Change in body weight during lockdown vs. current body weight among subjects *n* = 225.

During lockdown and remote work, 114 people (64.4%) noticed changes in eating behaviors: of which 99 people (44.0%) eat more than before lockdown, while 45 people (20.0%) eat less than before; 81 people (36.0%) believe they did not notice a change regarding the amount of food consumed. Changes in eating behavior did not correlate with body weight (*p* = 0.37), but did correlate with changes in body weight (*p* = 0.00000) (Figure 1). Body weight correlated with changes in body weight that occurred in the study group during lockdown (*p* = 0.000004) (Figure 2).

In a study by Błaszczyk-Bebenek et al. self-assessment of changes in the diet of respondents during the lockdown showed that the majority of the study group did not make any changes in their eating habits during the lockdown (32.4%) (27). In our study, 81 respondents (36.0%) declared that they did not change their eating behavior during the lockdown, 45 people (20.0%) said that they eat less than before, and 99 people (44.0%) said that they eat more than before. Sidor and Romski reported that 43.5% of the respondents declared higher food intake (8). In the same study, almost 40% of women did not observe any changes in body weight. In our study, as many as 55.5% of the subjects observed an increase in body weight, and only 21.3% decreased their body weight. In addition, a study by Ismail et al. (28) found that during the blockade, 32.8% claimed to have gained weight, 44.7% did not eat fruit daily, 35.3% did not eat vegetables daily, and 72.9% reported drinking less than eight glasses of water daily. In addition, there was a significant increase in the number of meals eaten per day, eating home-cooked meals, time spent sitting, stress, and sleep disturbance during the pandemic compared to before the pandemic. These results are the same as our own study, as lockdowns were also found to have a clear effect on the deterioration of eating habits among Silesian respondents - 55.5% increased their body weight and 44.0 changed their eating habits to worse than before the epidemiologic situation. Similar results have also been found in other studies, such as Changes in lifestyle, diet, and body weight during the first COVID 19 “lockdown” in a student sample, conducted by Palmer et al. (29).

### Eating Behavior During Remote Work During the COVID-19 Pandemic

When examining eating behaviors during remote work during the COVID-19 pandemic, no differences were observed between remote home office workers and hybrid workers. However, it is important to note that the studied eating behaviors during lockdown were not normal. There was a low intake of whole grain foods among remote workers (19.1% do not consume them daily). 22.7% consume more than the recommended amount of 500 g per week of red meat in various forms such as cold cuts, sausages, pate. The consumption of vegetables among the respondents was insufficient, only 31.1% declared the amount of 3–5 servings recommended by the National Center for Nutrition Education (21). It is noteworthy that 18.7% of the respondents do not consume vegetables and 23.6% of the fruit daily. Almost half of the respondents (48.4%) consume fish less frequently than once a week, and an adequate supply of fish is declared by only 10.7%, i.e., 2 or more times a week. Legume seeds are also consumed rarely, 68.0% eat them less than once a week. At the same time a significant proportion of the respondents eats sweets every day (30.2%), while fast-food dishes are rarely consumed, only 12.0% declare eating them several times a week, including 2 people who eat them every day (Table 2).

**Table 2:** Eating behaviors while working remotely during the COVID-19 pandemic (n=225).

**Meals consumed per day** ***n*** **(%)**	**Total**	**Home office**	**Hybrid**	***p*** **= value**
1–2 meals	12 (5.3%)	6 (4.4%)	6 (6.8%)	
3 meals	73 (32.5%)	42 (30.7%)	31 (35.2%)	0.38
4–5 meals	129 57.3(%)	84 (61.3%)	45 (51.2%)	
6 and more meals	11 (4.9%)	5 (3.6%)	6 (6.8%)	
**Frequency of consumption of whole grain cereals** ***n*** **(%)**				0.94
Less frequently than daily	43 (19.1%)	27 (19.7%)	16 (18.2%)	
1–2 times a day	143 (63.6%)	86 (62.8%)	57 (64.8%)	
3–5 times a day	39 (17.3%)	24 (17.5%)	15 (17.0%)	
**Frequency of consumption of red meat and meat products** ***n*** **(%)**				
I don't eat at all	34 (15,1%)	20 (14,6%)	14 (15,9%)	0.14
Not more than 500 g/week	140 (62,2%)	80 (58,4%)	60 (68,2%)	
More than 500 g/week	51 (22.7%)	37 (27.0%)	14 (15.9%)	
**Frequency of consumption of vegetables (excluding potatoes** ***n*** **(%)**				
Less frequently than daily	42 (18.7%)	30 (21.9%)	12 (13.6%)	0.24
1–2 times a day	113 (50.2%)	64 (46.7%)	49 (55.7%)	
3–5 times a day	70 (31.1%)	43 (31.4%)	27 (30.7%)	
**Frequency of fruit consumption** ***n*** **(%)**				
Less frequently than daily	53 (23.6%)	36 (26.3%)	17 (19.3%)	0.29
1 time per day	107 (47.6%)	63 (46.0%)	44 (50.0%)	
2–3 times a day	55 (24.4%)	30 (21.9%)	35 (28.4%)	
4 or more times a day	10 (4.4%)	8 (5.8%)	2 (2.3%)	
**Frequency of fish consumption** ***n*** **(%)**				
Less than once a week	109 (48.4%)	67 (48.9%)	42 (47.7%)	0.10
1 time per week	92 (40.9%)	51 (37.2%)	41 (46.6%)	
2 or more times a week	24 (10.7%)	19 (13.9%)	5 (4.7%)	
**Frequency of consumption of pulses** ***n*** **(%)**				
Less than once a week	153 (68.0%)	94 (68.6%)	59 (67.1%)	0.40
1 time per week	43 (19.1%)	23 (16.8%)	20 (22.7%)	
2 or more times a week	29 (12.9%)	20 (14.6%)	9 (10.3%)	
**Frequency of sweet tooth consumption** ***n*** **(%)**				
Once a week or less	79 (35.1%)	50 (36.5%)	29 (32.9%)	0.07
Several times a week	78 (34.7%)	40 (29.2%)	38 (43.2%)	
Every day	68 (30.2%)	47 (34.3%)	21 (23.9%)	
**Frequency of fast food consumption** ***n*** **(%)**				
Once a week or less	198 (88.0%)	120 (87.6%)	78 (88.7%)	0.90
Several times a week	25 (11.1%)	16 (11.6%)	9 (10.2%)	
Every day	2 (0.9%)	1 (0.7%)	1 (1.1%)	

*Home office—People working remotely from home in a home office*.

*Hybrid, People who work hybrid*.

An analysis by the United States Department of Agriculture found that food eaten outside the home was a contributing factor to poor diet quality, poor eating habits, and ultimately leads to overweight and obesity (30). According to WHO recommendations related to self-isolation at home during lockdown, eating at home reduced the frequency of contact with other people and reduced the likelihood of exposure to COVID-19 (31). However, in a study by Dragun et al., 27.3% of respondents increased their intake of sweets and snacks during lockdown (32). In a Canadian study by Carroll et al. more than half of the respondents felt that their diet had changed since COVID-19: the most common changes included eating more food (mothers 57%; fathers 46%; children 42%), eating more snacks (mothers 67%; fathers 59%; children 55%) and eating less fast food and/or take-out among parents (17). In a study by Deschasaux-Tanguy et al. 56.2% of participants reported that they modified their dietary behaviors during the blockade: changing their routine, spending more time cooking home-cooked meals, stopping eating out, having trouble maintaining a regular meal schedule, changes in food supply (buying less fresh produce, difficulty going to regular stores, difficulty finding preferred foods, difficulty buying organic foods (33). Among those surveyed during COVID-19, 60–70% of the students reported that they maintain the same eating habits, while about 20–38% of them reported an increased intake of fruits and vegetables (32), in a self-reported study, the intake of fruits and vegetables by the majority of the subjects is insufficient. It should also be noted that our study analyzed the behavior of working people, not students, which should also affect their dietary patterns, due to a larger budget allocated for nutrition than in the student group (Tables 3–4).

**Table 3:** Lifestyle while working remotely during the COVID-19 pandemic (*n* = 225).

**Hours of sleep per day:** ***n*** **(%)**	**Overall**	**Home office**	**Hybrid**	***p*** **= value**
Less than 7 h	83 (36.9%)	51 (37.2%)	32 (36.4%)	0.9
7–9 h	140 (62.2%)	85 (62.1%)	55 (62.5%)	
More than 9 h	2 (0.9%)	1 (0.7%)	1 (1.1%)	
**Physical activity during the day:** ***n*** **(%)**				
I do not engage in physical activity	43 (19.1%)	34 (34.8%)	9 (10.2%)	
Up to 30 min	98 (43.6%)	54 (39.4%)	44 (50.0%)	**0.02**
30–60 min	69 (30.6%)	38 (37.8%)	31 (35.2%)	
More than 60 min	15 (6.7%)	11 (8.0%)	4 (4.6%)	
**Frequency of alcohol consumption:** ***n*** **(%)**				
Does not consume	43 (19.1%)	25 (18.3%)	18 (20.5%)	
Occasionally	89 (39.6%)	53 (38.7%)	36 (40.9%)	0.20
Several times a month	59 (26.2%)	42 (30.6%)	17 (19.3%)	
Several times a week	25 (11.1%)	11 (8.0%)	14 (15.9%)	
Daily	9 (4.0%)	6 (4.4%)	3 (3.4%)	
**Cigarette smoking:** ***n*** **(%)**				
No	187 (83.2%)	113 (82.5%)	74 (84.1%)	
Yes, occasionally	19 (8.4%)	11 (8.0%)	8 (9.1%)	0.76
Yes	19 (8.4%)	13 (9.5%)	6 (6.8%)	

*Home office—People working remotely from home in a home office*.

*Hybrid—People who work hybrid*.

*Bold values are main statistical correlations*.

**Table 4:** Ratings of the emotional aspect of eating behaviors while working remotely during the COVID-19 pandemic (*n* = 225).

**TFEQ 13**		**Hybrid** ***n*** **(%)**	**Home office** ***n*** **(%)**	**Total** ***n*** **(%)**	***p*** **= value**
Food restrictions	Yes	61 (69.3%)	88 (64.2%)	149 (66.2%)	
	No	27 (30.7%)	49 (35.7%)	76 (33.8%)	0.43
No control over eating	No	67 (76.1%)	97 (70.8%)	164 (72.9%)	
	Yes	21 (23.9%)	40 (29.2%)	61 (27.1%)	0.78
Eating under the influence of emotions	No	50 (56.8%)	72 (52.6%)	122 (54.2%)	
	Yes	38 (43.2%)	65 (47.4%)	103 (45.8%)	0.22

### Lifestyle While Working Remotely During the COVID-19 Pandemic

Analyzing the lifestyle of the respondents, both the amount of sleep and frequency of alcohol consumption and cigarette smoking were similar among those working remotely from home on the so-called home office, and those working hybrid. Physical activity was more frequent among hybrid than home office workers (*p* = 0.02). However, it should be noted that as much as 19.1% of the entire study group did not undertake physical activity recommended by WHO (34), and 43.6% spent <30 min on it every day. Physical activity does not correlate with alcohol consumption (*p* = 0.80) or sleep (*p* = 0.068). There is also no correlation between cigarette smoking and alcohol consumption (*p* = 0.63). On the other hand, it is important to emphasize the fact that most of the respondents give up smoking cigarettes, 83.2% do not smoke.

The study by Błaszczyk-Bebenek et al. (27) estimated the frequency of alcohol consumption during blockade as follows 25.6% never consume, 26.0% consume 1–3 times a month, 19.9% once a week, 22.2% several times a week, 5.4% once a day and 1% several times a day. In self-reported alcohol consumption was lower, about 15% of respondents consume alcohol several times a week including daily.

Studies focusing on the impact of several weeks of reduced physical activity and modified food consumption have shown metabolic consequences (increased insulin resistance, inflammation, fat gain) even in the short term (35). In a French study, these adverse changes seemed to be related to being female, working at home and having children at home, i.e., parents who maintained their work activity while caring for their children (33).

### TFEQ 13 and Remote Working During the COVID-19 Pandemic

**Figure 3:**
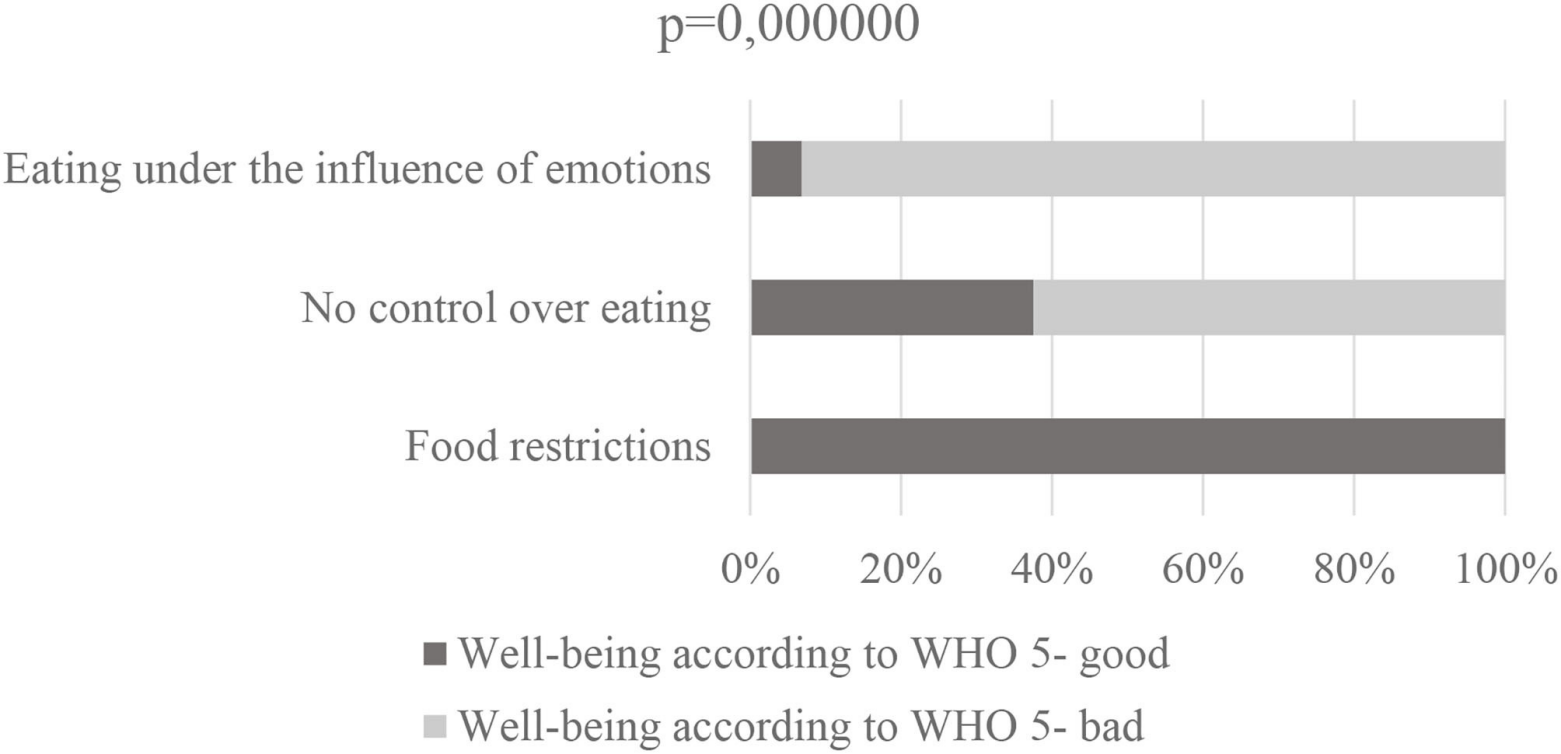
TFEQ 13, and well-being according to WHO-5 *n* = 225.

No relationship was observed between the form of remote work, the emotional aspect of eating behavior, both in terms of food restriction, no control over eating and eating under the influence of emotions (Figure 3, Table 5).

**Table 5:** Ratings of the emotional aspect of weight-related eating behaviors (*n* = 225).

**TFEQ 13**		**Underweight** ***n*** **(%)**	**Normowaga** ***n*** **(%)**	**Overweight** ***n*** **(%)**	**Obesity** ***n*** **(%)**	***p*** **= value**
Food restrictions	No	9 (100%)	89 (66.9%)	36 (67.9%)	15 (50.0%)	
	Yes	0 (0%)	44 (33.1%)	17 (32.1%)	15 50.0(%)	**0.04**
No control over eating	No	7 (77.8%)	99 (74.4%)	41 (77.4%)	17 (56.7%)	
	Yes	2 (22.2%)	34 (25.6%)	12 (22.6%)	13 (43.3%)	0.18
Eating under the influence of emotions	No	3 (33.3%)	79 (59.4%)	25 (47.2%)	15 (50.0%)	
	Yes	6 (66.7%)	54 (40.6%)	28 (52.8%)	15 (50.0%)	0.23

*Bold values are main statistical correlations*.

When analyzing the emotional aspect of eating behaviors vs. current body weight of the subjects, only eating restrictions were correlated with body weight (*p* = 0.04). Considering the correlations between weight change during lockdown—eating restrictions correlated with changes (*p* = 0.00567), as did no control over eating (*p* = 0.01), while no effect of weight change on emotional eating was observed (*p* = 0.7).

Individuals who restrict eating according to TFEQ 13 are more likely to report well-being (WHO-%), whereas individuals who are observed to lack control over eating and eat under emotional duress are more likely to report poor well-being (*p* = 0.000000) (Table 6).

**Table 6:** WHO well-being score −5, and form of remote working, body weight, and change in body weight during lockdown (*n* = 225).

**Form of work:** ***n*** **(%)**	**Well-being according to WHO 5- good**	**Well-being according to WHO 5- bad**	***p*** **= value**
Home office	48 (35.0%)	89 (65.0%)	0.88
Hybrid	30 (34.1%)	58 (65.9%)	
Total	78 (34.7%)	147 (65.3%)	
**Body weight:** ***n*** **(%)**			
Underweight	5 (55.5%)	4 (44.4%)	0.49
Normovaga	44 (33.0%)	89 (67.0%)	
Overweight	20 (37.7%)	33 (62.2%)	
Obesity	9 (30.0%)	21 (70.0%)	
**Change in body weight during lockdown:** ***n*** **(%)**			
Unchanged	26 (46.4%)	30 (53.6%)	
Decreased 1–5 kg	14 (48.3%)	15 (51.7%)	
Decreased 6–10 kg	6 (54.6%)	5 (45.4%)	**0.02**
Decreased 11–15 kg	1 (33.3%)	2 (66.7%)	
Decreased 16–20 kg	1 (100%)	0(%)	
Increased by 1–5 kg	24 (25.3%)	71 (74.7%)	
Increased 6–10 kg	6 (21.4%)	22 (78.6%)	
Increased by 11–15 kg	0 (%)	1 (100%)	
**Physical activity** ***n*** **(%)**			
Physical inactivity	10 (23.3%)	33 (76.7%)	
Up to 30 min	25 (25.5%)	37 (74.5%)	**0.0007**
30–60 min	34 (49.2%)	35 (50.7%)	
More than 60 min	9 (60.0%)	6 (40.0%)	

*Bold values are main statistical correlations*.

### WHO 5 a Remote Work During the COVID-19 Pandemic

In the study group, the mean % value obtained on the WHO-5 scale was 46.6% ± 22.0% (median 34.3%, min- 0%-max 100%)—in women 45.79%, men 49.7%, remote workers 45.1%, hybrid workers 48.8%. Analyzing mean WHO-5 scale values for body weight, underweight subjects had a mean value of 50.6%, normal weight 47.0%, overweight 45.7%, and obese 45.0%—differences between groups were not statistically significant.

There was no relationship between the form of remote work and the WHO 5 Index of Well-Being (*p* = 0.88), also there was no relationship between current body weight and WHO-5. However, it was observed that people who increased their body weight felt worse than those who decreased their body weight. However, it should be noted that despite weight reduction, most of the subjects had a poor well-being index. In the study, it was observed that physical activity affects well-being (*p* = 0.0007), the more physically active the subjects were the better they felt. Proper eating habits (including consumption of vegetables, fruits, fish, whole grain foods) did not affect well-being (*p* = 0.61), adequate amount of sleep also did not correlate with well-being (*p* = 0.20), as did alcohol consumption (*p* = 0.28).

The relationship between physical activity and mental health as found in a study by Pieh et al. is well-established, increased duration and intensity of physical activity were associated with a decreased prevalence of depression in men (36). In our study, this relationship was also confirmed.

Workers in many countries have been forced to work from home, with an estimated 38% of people having to stop going to their workplace due to the pandemic in China (37). People continue to report anxiety, worry about contracting the disease, and sleep problems during the COVID-19 pandemic, which is also reflected in a recent study showing that increased need for psychiatric support is reported by 80% of study participants (38). A review of studies on the impact of quarantine found that psychological effects of quarantine can include PTSD, depression, anxiety, sleep problems, increased fear, stigma, low self-esteem, and lack of self-control (39).

The literature shows that the coronavirus pandemic and anxiety associated with COVID-19 can disrupt workers' psychological well-being and hinder job performance. COVID-19 changed workers' traditional work situations and challenged their social situations. It may have made them feel lonely and potentially isolated from their work community. Previous research further suggests that individuals with neurosis may tend to respond more negatively to uncertainty and have poorer coping mechanisms in situations characterized by uncertainty. The increased use of technology has created additional stress and strain for many employees. From here, it is extremely important to take care of the mental health of individuals who are particularly vulnerable to mental disorders such as neurosis, anxiety, and depression (40).

### Limitations of the Study

One of the objectives of this study was to accurately examine health behaviors during lockdown and during remote work associated with it. Despite the large amount of work involved, there are some limitations of the present study that should be considered when evaluating and interpreting the results. The survey instrument, which was an electronic questionnaire, was more frequently completed by respondents with a university education due to the form of employment and the ability to work from home as this is likely to be computer-based work using internet connections.

The study was based on an anonymous and online survey, thus excluding the possibility of data verification. BMI was not measured by the researchers, but declared by the people surveyed. Therefore, it should be treated more as a rough estimate rather than an exact value. However, these limitations were insurmountable given the challenges of conducting such a survey during a nationwide quarantine. It should also be noted that the male subgroup was underrepresented, which is often the case in voluntary surveys, and also because the sample group may have represented occupational groups such as government officials, teachers, which in Poland make up the majority of these sectors of the economy.

In this study, a simplified approach was used to summarize the overall frequency of intake of food groups by relating them to the recommendations of the National Center for Nutrition Education, and the intake of each type of each food category was not distinguished. However, this was done to avoid any negative impact of the length and content of this questionnaire.

It is worth noting that standardized questionnaires such as TFEQ13 and WHO 5 were used in Polish language, which reduced the risk of bad translation of the questionnaire and, consequently, bad understanding by the respondents.

Further analyses would need to compare the health impacts also for workers who remained in their regular jobs. Such a group could have been used as a control group for the study, which would have enriched the methodology, although in the above study it was decided to develop results for the group directly affected in order to present its main criteria and make intra-group comparisons.

Finally, although this study provides an overview of eating behaviors during lockdown, its results cannot be interpreted in the context of the effects of long-term remote working, as this was not the purpose of this study. All of this suggests the need for careful interpretation of the data and calls for further research in this area of health behaviors while working remotely.

## Conclusions

- The study confirmed the change in dietary behavior and the occurrence of adverse health eating behaviors among remote and hybrid workers during the COVID-19 pandemic. At the same time, these changes correlated with the change in body weight of the subjects. Lifestyle analysis indicated no or little physical activity among 67.7% of the subjects.
- No relationship was observed between the form of remote work, the emotional aspect of eating behavior, both in terms of food restriction, lack of control over eating and eating under the influence of emotions.
- The study showed that factors such as physical exercise, proper eating habits, including the implementation of certain restrictions on food products had a positive impact on the well-being of the subjects.
- The results obtained due to the time limitation (lockdown time) cannot be interpreted in the context of the effects of long-term work. However, studies by multiple authors confirm that even short-term reductions in physical activity and irrational dietary modifications can produce metabolic consequences.

## Data Availability Statement

The original contributions presented in the study are included in the article/supplementary material, further inquiries can be directed to the corresponding author/s.

## Ethics Statement

Ethical review and approval was not required for this study in accordance with the local legislation and institutional requirements. The participants provided their informed consent to participate in this study.

## Author Contributions

ES and AB-D: conceptualization and investigation. AB-D, MG, ES, BC, and WS: methodology. AB-D and MG: data curation and writing—original draft preparation. AB-D, BC, and WS: writing—review and editing. ES: supervision. AB-D: project administration. All authors have read and agreed to the published version of the manuscript.

## Conflict of Interest

The authors declare that the research was conducted in the absence of any commercial or financial relationships that could be construed as a potential conflict of interest.

## Publisher's Note

All claims expressed in this article are solely those of the authors and do not necessarily represent those of their affiliated organizations, or those of the publisher, the editors and the reviewers. Any product that may be evaluated in this article, or claim that may be made by its manufacturer, is not guaranteed or endorsed by the publisher.

## Footnotes

^1^Polish Adaptation and Validation of The Physical Appearance Comparison Scale (PACS)—an Analysis Among Young People in Late Adolescence in The Context of Obesity: https://www.ncbi.nlm.nih.gov/pmc/articles/PMC8522941/.
